# Improving preimplantation genetic diagnosis (PGD) reliability by selection of sperm donor with the most informative haplotype

**DOI:** 10.1186/s12958-017-0247-4

**Published:** 2017-04-26

**Authors:** Mira Malcov, Veronica Gold, Sagit Peleg, Tsvia Frumkin, Foad Azem, Ami Amit, Dalit Ben-Yosef, Yuval Yaron, Adi Reches, Shimi Barda, Sandra E. Kleiman, Leah Yogev, Ron Hauser

**Affiliations:** 1 Wolfe PGD-Stem Cell Lab, Racine IVF Unit Lis Maternity Hospital, Tel Aviv Sourasky Medical Center, affiliated to the Sackler Faculty of Medicine, Tel Aviv University, Tel Aviv, Israel; 2 Prenatal Diagnosis Unit, Lis Maternity Hospital, Tel Aviv Sourasky Medical Center, affiliated to the Sackler Faculty of Medicine, Tel Aviv University, Tel Aviv, Israel; 3 The Institute for the Study of Fertility, Lis Maternity Hospital, Tel Aviv Sourasky Medical Center, affiliated to the Sackler Faculty of Medicine, Tel Aviv University, 6 Weizman Street, Tel Aviv, 6423906 Israel

**Keywords:** PGD, Sperm donor, Polymorphic markers, Haplotype

## Abstract

**Background:**

The study is aimed to describe a novel strategy that increases the accuracy and reliability of PGD in patients using sperm donation by pre-selecting the donor whose haplotype does not overlap the carrier’s one.

**Methods:**

A panel of 4–9 informative polymorphic markers, flanking the mutation in carriers of autosomal dominant/X-linked disorders, was tested in DNA of sperm donors before PGD. Whenever the lengths of donors’ repeats overlapped those of the women, additional donors’ DNA samples were analyzed. The donor that demonstrated the minimal overlapping with the patient was selected for IVF.

**Results:**

In 8 out of 17 carriers the markers of the initially chosen donors overlapped the patients’ alleles and 2–8 additional sperm donors for each patient were haplotyped. The selection of additional sperm donors increased the number of informative markers and reduced misdiagnosis risk from 6.00% ± 7.48 to 0.48% ±0.68. The PGD results were confirmed and no misdiagnosis was detected.

**Conclusions:**

Our study demonstrates that pre-selecting a sperm donor whose haplotype has minimal overlapping with the female’s haplotype, is critical for reducing the misdiagnosis risk and ensuring a reliable PGD. This strategy may contribute to prevent the transmission of affected IVF-PGD embryos using a simple and economical procedure.

**Trial registration:**

All procedures performed in studies involving human participants were in accordance with the ethical standards of the institutional and/or national research committee and with the 1964 Helsinki declaration and its later amendments or comparable ethical standards. DNA testing of donors was approved by the institutional Helsinki committee (registration number 319-08TLV, 2008). The present study was approved by the institutional Helsinki committee (registration number 0385-13TLV, 2013).

## Background

One of the options available for women who wish to have a child is to participate in a sperm donation program in order to conceive. When the woman is a carrier of a recessive genetic disease it is important to select a sperm donor that is free of the mutation in order to maximize the number of mutation free embryos following IVF-PGD. Notably, when the woman is a carrier of a dominant disorder or an X-linked disease, an affected offspring can be born regardless of the genetic status of the donor. Such consequences can be prevented by preimplantation genetic diagnosis (PGD). PGD entails the analysis of single cells that are biopsied from the preimplantation embryo and subjected to multiplex PCR [1–3]. Since the analyzed genetic material is extremely limited, this procedure is often accompanied by relatively high amplification failure, sample contamination and allele drop-out (ADO). ADO is probably the most disquieting limitation that can occurs on the single copy of either the normal or the mutated allele during the first of two PCR rounds. ADO and contamination can decrease the reliability of PGD and are considered to be the major causes of misdiagnosis [1, 4–6]. Highly sterile working area can prevent contamination and increasing denaturation temperature, optimizing PCR mixtures and calibrating the PCR program can dramatically reduce ADO, but it cannot be totally eliminated [7]. Additional attempts to prevent ADO include enlarging DNA amounts by biopsy of 5–10 trophectoderm cells from day 5 embryos [8]. However, the most efficient way to overcome those obstacles and prevent misdiagnosis caused by ADO is to co-amplify several informative polymorphic markers, most commonly CA-tandem repeats, flanking the tested mutation. This method utilizes the difference in the number of base pairs (bp) of the several adjacent repeats between those that are linked to the mutated allele or to the normal one. These differences are used to determine the haplotype and allow the discrimination between the alleles [9–13]. The reliability and accuracy of diagnosis increases proportionally with the number of informative markers: specifically, the greater the number of markers, the better will be the distinction between the normal vs. the abnormal alleles. The possibility of successfully implementing many polymorphic markers is determined by several factors: (1) the availability of these repeats in the vicinity of the tested gene, (2) the heterozygosity of the markers in the carrier female, and (3) “masking” of the repeats lengths by the partner’s alleles. This kind of masking or overlap between the genotypes dramatically reduces the informativity of the markers and may result in a suboptimal diagnosis due to insufficient data for the discrimination between healthy and affected embryos.

Whenever a woman who is a carrier of severe genetic disease uses her partner’s sperm and their haplotypes overlap, the PGD lab will invest precious time to search for further markers. One potential benefit of a carrier women who wish to conceive using a sperm donor is her possibility to choose, based on the donor’s characteristics she wishes, a genetically suitable donor whose haplotype does not overlap her haplotype. Therefore, the aim of the present study is to describe a novel strategy, which is relatively simple and cheap, enabling to increase the efficiency and accuracy of the PGD analysis by choosing the most genetically suitable sperm donor for carrier women.

## Methods

### Study population

A total of seventeen women known to be carriers of autosomal dominant or X-linked genetic disorders who required sperm donation and opted for PGD between the years 2008–2015 were recruited.

### Multiplex-PCR design

A tailor-made multiplex PCR composed of the direct detection of the familial mutation together with 6–12 polymorphic markers for haplotype determination, needed to be designed before carrying out PGD [11, 14]. The markers were retrieved from the literature and/or from genomic databases by searching 1.5 Mb upstream and 1.5 Mb downstream from the mutation site for mainly (CA)n or (TG)n repeats (where n >10). For the amplification of these loci, primers were designed using conventional databases (NCBI, UCSC) and Primer3 Plus software. Fluorescent primers were synthesized according to strict criteria (Sigma-Aldrich Co. Ltd). Polymorphic markers underwent amplification from genomic DNA in order to determine the extent of their informativity [15, 16].

### Haplotype analysis

The informativity of each polymorphic marker was determined in two stages. Firstly the carrier subject had to demonstrate heterozygosity for the analyzed markers. These markers were then screened on additional family members, whose carrier status is known, in order to determine the haplotypes that characterize the normal and the mutated alleles (“phasing”). The final informativity of the markers was determined by the inclusion of the donor’s values. All the possible parental haplotype combinations that were expected to be represented in the embryos were assessed. When the lengths of repeats differ, the best they contribute to the genetic analysis, while identical repeats length or a minimal difference of only one repeat (2 bp) is challenging to discern which allele is inherited to the embryo. Overlapping of the carrier’s normal alleles with the partner ones is undesirable since ADO of the mutated allele in the affected embryos might seem like healthy and transferable ones.

Consequently we have set a requirement standard of >2 bp difference between the couple’s lengths of markers linked to the normal allele for optimal allelic discrimination.

### Selection of sperm donors

When the original sperm donor was found to be unsuitable due to extensive overlapping of markers, up to 8 additional donors (who also met the woman’s personal preferences) were screened. The DNA of these donors was already available in the sperm bank lab since they had previously consented to DNA testing. Screening and characterizing the relevant markers in the additional candidates required a routine PCR reaction and amplicon analyses (few hours of routine bench work). The donor with maximal variation and minimal overlapping with the carrier patient’s markers was selected as the most suitable donor for future fertilization. The haplotype of embryos achieved by using the selected donor enabled the most accurate discrimination between normal and affected embryos in PGD cycles.

### Misdiagnosis risk calculation

In an attempt to assess the potential risk for misdiagnosis and to quantify the improvement achieved following donor substitution, we chose the ADO rate as the best predictor of risk for misdiagnosis with an opposite correlation to the number of available informative markers. Therefore, we calculated the maximal theoretical misdiagnosis risk as **0.15** ^**x**^, where 0.15 is the maximal ADO rate empirically observed in our lab - up to 15% for each locus, and ***x*** is the total number of informative tested markers (in cases where the mutation site is also polymorphic throughout the population, this site is counted too, like in Huntington disease). Consequently, the misdiagnosis risk for every woman’s PGD will decrease as the number of informative markers will increase, which clearly will vary upon the donor’s haplotype. In X-linked disorders the overlapping risk is relevant only for female embryos since male embryos inherit the paternal Y chromosome.

### Single cells analysis

The molecular diagnosis setup was initially evaluated on single leukocytes isolated from peripheral blood of normal, carrier and affected individuals and it comprised a multiplex-nested PCR. The familial mutation, polymorphic markers and gender determination loci in X-linked diseases, were amplified.

For the first round PCR, the following were added to the reaction tube containing the single cell in alkaline lysis buffer: 2 μl of 10× PCR buffer (OptiBuffer, Bioline), 1 μl MgCl2 (50 mM), 2 μl of 5× Specificity Enhancer (Bioline), 6 μl H2O, 0.5 μl tricine 1 M, 1 μl of dNTP mixture stock solution (5 mM), 1 μl of DMSO,0.5 μl gelatin (1% w/v) and 0.5 μM of each primer. The mixture was heated to 96 °C for 8 min for extended denaturation and temperature was then decreased to 75 °C. At this stage, 5 μl of enzyme mix containing 0.5 μl 10× PCR buffer (OptiBuffer, Bioline), 0.25 μl MgCl2 (50 mM), 0.25 μl *Taq* polymerase (Bio-X-Act, 4u/ml) and 4 μl H20 was added. Amplification began with a single denaturation step at 98 °C for 2 min, followed by ten cycles of denaturation at 96 °C for 1 min, annealing at 60 °C for 2 min and extension at 72 °C for 3 min. This was followed by six more cycles of denaturation at 94 °C for 45 s, annealing at 60 °C for 1 min and extension at 72 °C for 3 min. Final extension was performed at 72 °C for 8 min. For the second round PCR (“nested PCR”), 1 μl of the first round PCR product was added into separate PCR tubes for each of the loci. The following were added to every tube: 5 μl of 5× PCR buffer (MyFi Reaction Buffer, Bioline) 0.5 μl of MyFi DNA Polymerase (2U/μl), 1 μl of each primer (from 20 μM stocks) and H2O was added for a final volume of 25 μl. The mixtures underwent prolonged denaturation at 96 °C for 8 min and a single denaturation step of 98 °C for 2 min, followed by 14 cycles of denaturation at 96 °C for 1 min, annealing at 60 °C for 2 min and extension at 72 °C for 2 min. This was followed by 20 more cycles of denaturation at 94 °C for 45 s, annealing at 60 °C for 1 min and extension at 72 °C for 2 min. Final extension was performed at 72 °C for 8 min Subsequently, fluorescent PCR products were analyzed on the Applied Biosystems 3130xl Genetic Analyzer using GeneMapper® v4.0 software (“GeneScan® analysis”), while enzymatic restriction and electrophoresis were generally used for mutation analysis [17, 18].

The application of this diagnosis setup onto blastomeres for PGD analysis was restricted to reactions demonstrating high accuracy as assessed by amplification rate > 98% and allele dropout (ADO) rate <15% for each locus. The system should also confirmed no false negatives or false positives in at least 10 single leukocytes [18–20].

### Prevention of contamination

To avoid contamination, the first round single-cell PCR was performed in an isolated sterile room that is air-filtered, under constant positive pressure, and separate from the IVF and the molecular labs. Lysis buffer and PBS were UV irradiated for 1 h.

### IVF-PGD procedure

For PGD, the carrier women underwent a standard in vitro fertilization (IVF) procedure, which entails controlled ovarian hyperstimulation using gonadotrophins, oocytes retrieval at 35–36 h after administration of human chorionic gonadotrophins, and fertilization of denuded oocytes by intracytoplasmic sperm injection (ICSI) [21]. Fertilization was determined 18–20 h after ICSI by the presence of 2 pronuclei. A biopsy of cleaved embryos was performed by means of a micromanipulator (Narishige, Japan) mounted on an inverted microscope (Nikon eclipse TE 200). The zona pellucida of day 3 embryos was perforated using in-contact laser apparatus (ZILOS, Hamilton), and 1 or 2 cells were aspirated [22]. The number of biopsied blastomeres is depended upon embryo cleavage rate and estimated risk of misdiagnosis (i.e., inheritance pattern and the number of informative markers).

Biopsied blastomeres were washed, transferred to 0.2 ml sterile PCR tubes and heated for DNAse inactivation. These single cells were then kept at −20°C prior to the PCR analysis. A multiplex-nested PCR protocol for single cells was applied on blastomeres and control single leukocytes, as described above [11, 23].

## Results

Seventeen female carriers of diverse autosomal dominant or X-linked genetic disorders entered the sperm donor program (Table 1). Initially, donor sperm was selected according to the woman’s personal preferences, independent to its haplotype.

**Table 1:** Genetic etiology for PGD in female patients utilizing sperm donation

Disease	Inheritance pattern	No. of patients
Fragile X	X-linked	3
DMD	X-linked	2
Alport syndrome	X-linked	1
Norrie	X-linked	1
NF1	Autosomal dominant	2
Retinoblastoma	Autosomal dominant	1
Marfan syndrome	Autosomal dominant	1
BRCA2	Autosomal dominant	1
Kallman syndrome	Autosomal dominant	1
Huntington disease	Autosomal dominant	2
MD2	Autosomal dominant	1
Achondroplasia	Autosomal dominant	1
Total		17

DMD = Duchene muscular dystrophy, NF1 = neurofibromatosis, BRCA = breast cancer, MD2 = myotonic dystrophy 2

The haplotype of each woman was established by screening a panel of 6–12 polymorphic markers flanking the specific mutation. The markers that showed heterozygosity were defined as potentially informative, explicitly; they contribute to discriminate between the normal and mutant alleles. This preliminary analysis demonstrated 4–9 informative markers for the 17 tested patients, a number that is considered sufficient for reliable diagnosis. These markers were further characterized on DNA from chosen donors and overlapping extent was assessed. Table 2 presents the number of informative markers for each of the 17 study women, an average of 5.82 ± 1.67 markers, compared to the number of informative markers after taking the overlapping of the donor’s alleles into consideration, an average of 2.76 ± 1.30 markers. In 3 cases, the initially selected donor was satisfying (i.e., the donors’ haplotype did not overlap significantly or at all the patients’ haplotype). Another 6 women insisted upon their first choice for donor, and treatment continued following the characterization and utilization of new polymorphic markers that were designed and ordered considering their particular genotypes constitutions. This workup demanded additional cost and caused a delay in treatment. Still, in 8 cases (47% of the patients), the donor’s corresponding markers extensively overlapped the patient’s ones demonstrating a relatively high potential for misdiagnosis risk of 6% (Table 4). These carrier women consented to replace the donor, thus the DNA from additional sperm donors was screened in relation to the informative markers of these women until a suitable one, i.e., with the least overlapping haplotype, was found. As an example, the haplotype of a patient affected with autosomal dominant Huntington disease, Patient #17, and haplotypes of several tested sperm donors, are shown in Table 3. It can be noticed that initially 9 markers were found to be informative. However, following the combination with the firstly chosen donor (Donor 1) 5 out of 9 markers, linked to the normal allele, were masked and turned to be uninformative. Moreover, the patient’s normal allele in the mutation loci was totally overlapped by both alleles of the donor. This given scenario increased the potential risk for misdiagnosis and prevented the ability to positively demonstrating the transmission of the patient’s normal allele to the offspring. Focusing on this late parameter, the 4 additional tested donors were significantly more genetically-suitable. Out of them, “Donor 5” was informative for all the 9 markers including the mutation site, bringing back the potential misdiagnosis risk to infinitesimal value (0.0004%). The patient agreed to exchange the first choice with donor 5 and it was further used for fertilization.

**Table 2:** Comparison of number of informative polymorphic markers before and after donors’ contribution

Patient #	Inheritance pattern	No. of informative markers in patient	No. of informative markers following donor contribution
1	X-linked	4	4
2	X-linked	5	3
3	X-linked	6	3
4	X-linked	7	5
5	X-linked	6	2
6	X-linked	8	4
7	X-linked	6	1
8	Auto. dom.	5	2
9	Auto. dom.	5	2
10	Auto. dom.	5	5
11	Auto. dom.	4	2
12	Auto. dom.	3	1
13	Auto. dom	5	1
14	Auto. dom.	9	3
15	Auto. dom.	6	2
16	Auto. dom.	6	3
17	Auto. dom.	9	4
Average number of informative markers		5.82 ± 1.67	2.76 ± ±1.30

Auto. dom. = autosomal dominant

The influence of the donor in X-linked disorders is relevant only for female embryos, since male embryos inherit Y paternal chromosome

**Table 3:** Screening various sperm donors’ genotypes for a representative PGD patient with Huntington disease

Markers	*chr4:* 1,953,007–1,953,486^b^	*chr4:* 2,351,587–2,352,006	*chr4:* 3,038,646– 3,039,039	*chr4:* 3,052,695–3,053,077	*chr4:* 3,076,297–3,076,716	*chr4:* 3,085,088–3,085,500	*chr4:* 3,325,450–3,325,833	*chr4*: 3,230,879–3,231,097	*chr4*: 3,377,889–3,378,176
Patient #17	231/**235**	**235**/239	**390**/396	**381**/389	276/**337**	271/**281**	373/**377**	**189**/204	**419**/428
Donor 1	235/240	235/235	*390/396*	*381/389*	*276/276*	269/281	*375/375*	*189/204*	419/421
Donor 2	227/241	*233/239*	*394/396*	*387/391*	283/286	277/284	*375/378*	*189/204*	402/419
Donor 3	227/241	229/235	389/391	381/385	280/286	*271/277*	*373/375*	*206/208*	402/421
Donor 4	*220n231*	233/233	394/400	385/387	286/286	277/285	*369/373*	*204/206*	423/421
Donor 5^a^	222/222	233/233	390/400	383/385	280/286	283/285	369/377	173/189	419/421

The numbers represent the length of amplified markers in bp, as read by a fragment analyzer

The bold numbers represent the markers’ lengths linked to the mutant allele

The donor’s alleles that differ from the subject’s normal alleles by ≤2 bp (considered as “overlapping”) are written in *italics*. Alleles’ differences >2 bp. in normal alleles (not overlapping) are underlined

^a^Selected as the most suitable donor

^b^Based on UCSC Genome Browser on Human Feb. 2009 (GRCh37/hg19) Assembly

Table 4 shows the numbers of informative markers and the calculated misdiagnosis risks for the 8 women where the first donor’s haplotype dramatically overlapped their markers and impaired the accuracy and reliability of the diagnosis. These numbers are compared to the number of markers and the re-calculated misdiagnosis risk after choosing a best matching sperm donor’s haplotype. Average of 4.25 ± 1.75 additional donors per woman (a total of 34 donors) were haplotyped for the 8 women, increasing the average number of informative markers from 2.38 ± 1.30 to 5.0 ± 1.93. This strategy led to a significant reduction in the general misdiagnosis risk from 6.00 ± 7.48% to a maximum of 0.48 ± 0.68% (Table 4).

**Table 4:** Increasing the accuracy of genetic diagnosis following selection of additional donors

	First chosen donor		Total no. of additional tested donors	Newly selected donor	
Subject No.	No. of informative markers	Misdiagnosis risk		No. of informative markers	Misdiagnosis risk
#6	4	0.05% in female offspring	3	5	0.10%
#7	1	15% in female offspring	4	5	0.10%
#12	1	15%	4	3	1.56%
#13	1	15%	5	3	1.56%
#14	3	0.34%	8	5	0.10%
#15	2	2.25%	2	6	0.02%
#16	3	0.34%	4	4	0.39%
#17	4	0.0506%	4	9	0.0004%
Average ± SEM	2.38 ± 1.30	6.00% ±7.48	4.25 ± 1.75	5.00 ± 1.93	0.48% ±0.68

## Discussion

It is currently estimated that over 10,000 of human diseases are known to be monogenic. The global prevalence of all single gene diseases at birth is approximately 1/100 (WHO website, Genomic resource center, Genes and human disease, Monogenic diseases, http://www.who.int/genomics/public/geneticdiseases/en/index2.html) and one of the main objectives of fertility treatment is to avoid transmitting genetic disorders to the offspring. Women desiring to utilize the sperm donor program in Israel are requested to undergo genetic screening according to the recommendations of the Israeli Genetic Association, which are based on the prevalence of genetic diseases related to ethnic origins. Non-carrier women can choose donors according to specific ethnic origin, physical characteristics, occupation, fields of interest, etc. If the woman is found to be carrier of a recessive disorder, she can choose among sperm donors that had been tested for the common mutations of that particular gene. The Israeli Ministry of Health requires genetic testing solely of Tay-Sachs mutations in donor sperm, although most of the sperm banks in Israel do test donors for a variety of other genetic disorders.

Donors in the USA are selected according to the “Recommendations for gamete and embryo donation”. Donors should not have any major Mendelian disorder nor should they have any significant familial disease with a major genetic component [24]. A survey revealed that the genetic testing performed on sperm donors varies significantly at sperm banks across the United States [25].

For carriers of dominant or X-linked diseases undergoing PGD, the genetic status of the donor is irrelevant regarding genetic offspring’s outcome; nevertheless, the variability of polymorphic markers lengths flanking the mutated gene, which is never tested by routine, can significantly affect the reliability of the PGD analysis and influence the misdiagnosis risk.

In order to prevent overlapping of donor’s polymorphic markers with the carrier’s one, the PGD lab can opt for diagnosis of the maternal genetic material only, by Polar body (PB) biopsy [26, 27]. This is achieved by sequential biopsy of the first and second PB discarded from the maturing oocyte in the end of the first and second meiosis, respectively. To deduce the maternal contribution to the developing zygote, the genetic constitutions of the first and second PB should be eliminated from the initial genome composition of the primary oocyte (2n, 4C). This turns to be disadvantaging compared to the diagnosis of blastomeres or trophectoderm biopsy where the embryo genetic status is directly diagnosed instead of being deduced. Yet, ADO events can jeopardize the results and frequently an additional biopsy of the embryos is required, which turns the PB diagnosis into highly complex and exhausting for IVF and PGD labs [28]. After practicing this approach for several years at our unit, it has been decided not to opt for it unless it is inevitable (for example with *de novo* maternal mutations). Consequently, the available options for prevention of errors in diagnosis caused by ADO are to use several informative markers or enlarging the available embryonic DNA amount. The last can be attained by the biopsy of 5–10 trophectoderm cells 5 days following fertilization, at the blastocyst stage [8]. This approach nowadays constitutes a considerable proportion of PGD biopsies, however it should be noticed that not all cleavage stage embryos will eventually reach blastocyst stage and that prolonged incubation can affect epigenetic patterns and may have detrimental effects on offspring health [29–31]. Additionally, the remaining time for the molecular analysis before hatching of biopsied embryos is completed is restricted. Most labs will freeze the embryos, each one separately, immediately following biopsy and transfer the healthy ones in the next thawing cycle [32]. Contemplating all the above mentioned considerations, we choose to combine day 3 biopsy benefits with enlargement of the available informative polymorphic markers.

Bioinformatics search for the identification and localization of at least dozen repeats, following by primers ordering, can extend several working days and two more weeks till the primers are supplied. The price for each fluorescence primer pairs is around US$300, taking into consideration that longer primers and higher purification scales are needed for efficient amplification in single-cell PCR. Overall, adding 12 polymorphic markers to the analysis that will result in the addition of 4–7 informative ones will cost around US$3600 and delay the setup for around one additional month. When sperm donor is employed, instead of expanding the polymorphic marker panel, the same reliability and accuracy can be achieved by selecting the most genetically suitable sperm donor. Screening multiple sperm donors for the specific DNA loci, represent the patient’s informative markers, is a rapid, simple and conclusive procedure with instantaneous effect. To the best of our knowledge, it is the first time this protocol for sperm donor selection has been proposed for PGD patients.

In 14 out of the 17 cases in our program, the first chosen donors increased the calculated misdiagnosis risk. Replacing the first choice with a donor that shared the minimal number of overlapping lengths of markers (mainly in the normal allele) dramatically raised the reliability in 8 cases. Routine reanalysis of non-transferred diagnosed embryos as well as prenatal tests confirmed PGD results and no misdiagnosis had been demonstrated.

## Conclusions

We present a novel and simple strategy aimed at minimizing the risk of misdiagnosis in PGD for carrier women by means of a meticulous selection of sperm donor. It can be applied for every single gene disorder and chromosomal rearrangements as long as the diagnosis is performed by haplotype analysis based on polymorphic marker repeats.

Whenever it is feasible to genetically test donors and identify the one that demonstrates the least overlapping of haplotypes with those of the carrier, it would enable bypassing the tedious and expensive task of screening for additional informative polymorphic markers. These “best matching haplotypes” mean the maximal differentiation between the donor’s alleles and the alleles of the carrier female, which, in turn, signifies a better chance of preventing the transfer of embryos affected with severe inherited disorders in the setting of an assisted reproduction program followed by PGD. Cooperative efforts on the part of the PGD lab with the sperm bank made this strategy feasible and it is now routinely used in our PGD setup and is applicable for all inherited genetic disorders.

## References

[1] Ray PF, Handyside AH (1996). PCR from single cells for preimplantation diagnosis. Methods Mol Med.

[2] Sermon K, De Vos A, Van de Velde H, Seneca S, Lissens W, Joris H, Vandervorst M, Van Steirteghem A, Liebaers I (1998). Fluorescent PCR and automated fragment analysis for the clinical application of preimplantation genetic diagnosis of myotonic dystrophy (Steinert’s disease). Mol Hum Reprod.

[3] Verlinsky Y, Rechitsky S, Cieslak J, Ivakhnenko V, Wolf G, Lifchez A, Kaplan B, Moise J, Walle J, White M (1997). Preimplantation diagnosis of single gene disorders by two-step oocyte genetic analysis using first and second polar body. Biochem Mol Med.

[4] Piyamongkol W, Bermudez MG, Harper JC, Wells D (2003). Detailed investigation of factors influencing amplification efficiency and allele drop-out in single cell PCR: implications for preimplantation genetic diagnosis. Mol Hum Reprod.

[5] Wells D (2004). Advances in preimplantation genetic diagnosis. Eur J Obstet Gynecol Reprod Biol.

[6] Wilton L, Thornhill A, Traeger-Synodinos J, Sermon KD, Harper JC (2009). The causes of misdiagnosis and adverse outcomes in PGD. Hum Reprod.

[7] Ray PF, Handyside AH (1996). Increasing the denaturation temperature during the first cycles of amplification reduces allele dropout from single cells for preimplantation genetic diagnosis. Mol Hum Reprod.

[8] Chang LJ, Huang CC, Tsai YY, Hung CC, Fang MY, Lin YC, Su YN, Chen SU, Yang YS (2013). Blastocyst biopsy and vitrification are effective for preimplantation genetic diagnosis of monogenic diseases. Hum Reprod.

[9] Chen M, Chan JK, Nadarajah S, Tan AS, Chan ML, Mathew J, Saw EE, Lim C, Wong W, Cheah FS (2015). Single-tube nonaplex microsatellite PCR panel for preimplantation genetic diagnosis of Hb Bart’s hydrops fetalis syndrome. Prenat Diagn.

[10] Fiorentino F, Biricik A, Nuccitelli A, De Palma R, Kahraman S, Iacobelli M, Trengia V, Caserta D, Bonu MA, Borini A, Baldi M (2006). Strategies and clinical outcome of 250 cycles of Preimplantation Genetic Diagnosis for single gene disorders. Hum Reprod.

[11] Malcov M, Naiman T, Yosef DB, Carmon A, Mey-Raz N, Amit A, Vagman I, Yaron Y (2007). Preimplantation genetic diagnosis for fragile X syndrome using multiplex nested PCR. Reprod Biomed Online.

[12] Renbaum P, Brooks B, Kaplan Y, Eldar-Geva T, Margalioth EJ, Levy-Lahad E, Altarescu G (2007). Advantages of multiple markers and polar body analysis in preimplantation genetic diagnosis for Alagille disease. Prenat Diagn.

[13] Renwick PJ, Lewis CM, Abbs S, Ogilvie CM (2007). Determination of the genetic status of cleavage-stage human embryos by microsatellite marker analysis following multiple displacement amplification. Prenat Diagn.

[14] Altarescu G, Brooks B, Kaplan Y, Eldar-Geva T, Margalioth EJ, Levy-Lahad E, Renbaum P (2006). Single-sperm analysis for haplotype construction of de-novo paternal mutations: application to PGD for neurofibromatosis type 1. Hum Reprod.

[15] Bachinski LL, Udd B, Meola G, Sansone V, Bassez G, Eymard B, Thornton CA, Moxley RT, Harper PS, Rogers MT (2003). Confirmation of the type 2 myotonic dystrophy (CCTG)n expansion mutation in patients with proximal myotonic myopathy/proximal myotonic dystrophy of different European origins: a single shared haplotype indicates an ancestral founder effect. Am J Hum Genet.

[16] Cabral WA, Barnes AM, Adeyemo A, Cushing K, Chitayat D, Porter FD, Panny SR, Gulamali-Majid F, Tishkoff SA, Rebbeck TR (2012). A founder mutation in LEPRE1 carried by 1.5% of West Africans and 0.4% of African Americans causes lethal recessive osteogenesis imperfecta. Genet Med.

[17] Girardet A, Ishmukhametova A, Willems M, Coubes C, Hamamah S, Anahory T, Des Georges M, Claustres M (2015). Preimplantation genetic diagnosis for cystic fibrosis: the Montpellier center’s 10-year experience. Clin Genet.

[18] Malcov M, Schwartz T, Mei-Raz N, Yosef DB, Amit A, Lessing JB, Shomrat R, Orr-Urtreger A, Yaron Y (2004). Multiplex nested PCR for preimplantation genetic diagnosis of spinal muscular atrophy. Fetal Diagn Ther.

[19] Dreesen J, Destouni A, Kourlaba G, Degn B, Mette WC, Carvalho F, Moutou C, Sengupta S, Dhanjal S, Renwick P (2014). Evaluation of PCR-based preimplantation genetic diagnosis applied to monogenic diseases: a collaborative ESHRE PGD consortium study. Eur J Hum Genet.

[20] Malcov M, Ben-Yosef D, Schwartz T, Mey-Raz N, Azem F, Lessing JB, Amit A, Yaron Y (2005). Preimplantation genetic diagnosis (PGD) for Duchenne muscular dystrophy (DMD) by triplex-nested PCR. Prenat Diagn.

[21] Gold RS, Azem F, Yovel I, Wagman I, Amit A, Lessing JB (2000). Does ICSI affect early serum beta-HCG in pregnancies achieved after IVF?. Hum Reprod.

[22] De Vos A, Staessen C, De Rycke M, Verpoest W, Haentjens P, Devroey P, Liebaers I, Van de Velde H (2009). Impact of cleavage-stage embryo biopsy in view of PGD on human blastocyst implantation: a prospective cohort of single embryo transfers. Hum Reprod.

[23] Yaron Y, Schwartz T, Mey-Raz N, Amit A, Lessing JB, Malcov M (2005). Preimplantation genetic diagnosis of Canavan disease. Fetal Diagn Ther.

[24] Practice Committee of American Society for Reproductive M (2013). Practice Committee of Society for Assisted Reproductive T: Recommendations for gamete and embryo donation: a committee opinion. Fertil Steril.

[25] Sims CA, Callum P, Ray M, Iger J, Falk RE (2010). Genetic testing of sperm donors: survey of current practices. Fertil Steril.

[26] Altarescu G, Renbaum P, Brooks PB, Margalioth EJ, Ben Chetrit A, Munter G, Levy-Lahad E, Eldar-Geva T (2008). Successful polar body-based preimplantation genetic diagnosis for achondroplasia. Reprod Biomed Online.

[27] Verlinsky Y, Rechitsky S, Verlinsky O, Kenigsberg D, Moshella J, Ivakhnenko V, Masciangelo C, Strom C, Kuliev A (2002). Polar body-based preimplantation diagnosis for X-linked disorders. Reprod Biomed Online.

[28] Levin I, Almog B, Shwartz T, Gold V, Ben-Yosef D, Shaubi M, Amit A, Malcov M (2012). Effects of laser polar-body biopsy on embryo quality. Fertil Steril.

[29] Bazrgar M, Gourabi H, Eftekhari-Yazdi P, Vazirinasab H, Fakhri M, Hassani F, Chehrazi M, Valojerdi MR (2016). The Effect of Prolonged Culture of Chromosomally Abnormal Human Embryos on The Rate of Diploid Cells. Int J Fertil Steril.

[30] Iliadou AN, Janson PC, Cnattingius S (2011). Epigenetics and assisted reproductive technology. J Intern Med.

[31] Pinborg A, Loft A, Romundstad LB, Wennerholm UB, Soderstrom-Anttila V, Bergh C, Aittomaki K (2016). Epigenetics and assisted reproductive technologies. Acta Obstet Gynecol Scand.

[32] Dahdouh EM, Balayla J, Garcia-Velasco JA (2015). Impact of blastocyst biopsy and comprehensive chromosome screening technology on preimplantation genetic screening: a systematic review of randomized controlled trials. Reprod Biomed Online.

